# Repeated CyberKnife stereotactic body radiation therapy in hepatocellular carcinoma

**DOI:** 10.1186/s13014-020-1457-z

**Published:** 2020-01-09

**Authors:** Jing Sun, Can Ouyang, Xiaoyun Chang, Aimin Zhang, Quan Wang, Wengang Li, Dan Zhang, Jia Wang, Dong Li, Xuezhang Duan

**Affiliations:** Department of Radiation Oncology, The Fifth Medical Center of PLA General Hospital (302 Military Hospital), Beijing, 100039 China

**Keywords:** Hepatocellular carcinoma, Second-course radiotherapy, Stereotactic body radiation therapy, Prognosis

## Abstract

**Background:**

To explore the survival and side effects of repeated CyberKnife stereotactic body radiation therapy (CK-SBRT) on hepatocellular carcinoma patients.

**Methods:**

24 HCC patients were collected at The Fifth Medical Center of PLA General Hospital from November 2011 to July 2016. They received second-course CK-SBRT with a prescribed dose of 50(48–55) Gy/5-8fx, and a single dose of 10 (7–11) Gy/fx. Cumulative overall survival rates (OS), progression-free survival rates (PFS) and local control rates (LC) were calculated by Kaplan-Meier method.

**Results:**

All patients finished their radiotherapy plans. The 1-,2- and 3-year cumulative OS rate were 95.8,81.1 and 60.8%. The 1-,2- and 3-year LC rate were 95.5,90.7 and 90.7%, respectively. The 1-, 2- and 3-year PFS were 74.8, 49.2 and 39.4%, respectively. 16 patients complained of fatigue during second-course therapy, 2 patients showed Grade 2 gastrointestinal reaction, 1 patient was diagnosed radiation-induced liver disease and none died. PFS was significantly higher in the interval time < 12 months group than in the interval time ≥ 12 months group (*p* = 0.030).

**Conclusions:**

It is preliminarily believed that re-CK-SBRT is an effective and safe treatment for HCC patients, but the treatment criteria should be strictly controlled.

## Background

Hepatocellular carcinoma (HCC) is the sixth most common cancer worldwide and the fourth most common cause of cancer death [1]. In addition to the curative therapies (liver resection, liver transplantation, radiofrequency ablation, etc), stereotactic body radiation therapy (SBRT) also achieved a satisfactory local control rate in treating HCC patients, and provided a new option for patients. Numerous studies showed SBRT was an effective method for HCC patients with different stages and tumor sizes [2–5].

Relapse and metastasis were the most important causes affecting HCC patients’ prognosis. When relapse or metastasis occurred, radiofrequency ablation (RFA) and trans-arterial chemo embolism (TACE) were often adopt for these patients, and liver re-resection were occasionally applied to the patients whose indocyanine green (ICG) test’s results were feasible [6]. However, for HCC patients with first-course SBRT, is repeated SBRT safe and effective for their tumor relapsing? Until now, there has little study on this field.

## Methods

### Patients selection

The eligibility criteria: (1) relapse and metastasis in liver were confirmed through image examination and laboratory tests. (2) Child-Pugh A or B classification; (3) ECOG PS 0–1 score; (4) white blood count≥2*10^9^/L, platelet count≥60*10^9^/L; (5) the period between first-course CyberKnife (Accuray, USA) SBRT (CK-SBRT) and second-course CK-SBRT was more than 6 months (6) the distance between lesion and organ at risks (especially skin, stomach, duodenum, colon and bowel) was equal or greater than 10 mm.

The excluded criteria: (1) patient with RILD during first-course CK-SBRT; (2) intractable ascites; (3) with extrahepatic metastasis; (4) the range of lesion was not confirmed by image examination; (5) with serious internal medicine disease; (6) combined with other therapies, including molecular targeted treatment and anti-PD-1 therapy, etc. (7) normal effective liver volume<700 cc.

### Patients’ general characteristics

There were 24 HCC cases with relapse or metastasis underwent second-course CK-SBRT in The Fifth Medical Center of PLA General Hospital between November 2011 and July 2016. Among them, eighteen patients were male, six were female. The median ages were 53 (42–77) years old. The average of tumor diameter was 2.5 (0.8–3.5) cm. Alpha-fetoprotein (AFP) value was 7.16 (2.25–514) ng/ml. Twenty-one patients were Child-Pugh A classification, and three patients were Child-Pugh B. Nineteen cases were with chronic hepatitis B and five were with chronic hepatitis C. The prescribed dose of first-course CK-SBRT were 50(48–56) Gy/5-8fx. Their interval period between first-course CK-SBRT and second-course CK-SBRT was 21(6–53) months. Among these patients, three patients were primary lesions, and twenty-one patients were new lesions. Among the patients with new lesions, 4 patients had lesions in the same liver segmentation, and 17 patients in different liver segmentation (according to Couinaud segmentation). The characteristics of patients were shown in Table 1.

**Table 1:** Clinical and biochemical characteristics of patients enrolled in this study

Variables	n
Gender	
Male	18
Female	6
Age (years)	
median	53
range	42–77
Type of chronic hepatitis	
Hepatitis B	19
Hepatitis C	5
Child-Pugh classification	
A	21
B	3
Maximum diameter of tumor (cm)	
median	2.5
range	0.8–3.5
AFP (ng/ml)	
median	7.16
range	2.25–514
Previous treatment of lesions	
Yes	3
No	21

*AFP* alpha-fetoprotein

### Therapeutic method

Before second-course CK-SBRT, we should confirm the positional relation between fiducial marker and lesion. During CK-SBRT treatment, the CK tracks the tumor by tracking fiducials to confirm the relative position of the fiducial marker and the tumor in the synchrony system. Therefore, the fiducial markers (at least three ones) were needed to place around the tumor, and the distance between all the markers and the tumor was generally no more than 6 cm. If all or part of the markers cannot be used, different amounts of fiducial marker should be implanted to make sure at least three markers can be used for CK-SBRT tracking of new lesions during the treatment. There were 13 patients in this study receiving complementary fiducial marker implantation. GTV (gross tumor volume) was defined as tumor in image examination, which extended 2-5 mm was defined PTV (plan target volume). The prescribed doses were 50(48–55) Gy/5-8fx. The normal tissue tolerance doses were limited according to AAPM TG-101 [7]. The radiation schedules and parameters were shown in Table 2.

**Table 2:** Radiation schedules and parameters

	First SBRT course								Second SBRT course						Interval time (months)	Previous treatment of lesions
Patients no.	Total dose (Gy)	Single dose (Gy)	BED_10_ (Gy)	Volume of GTV	Residual liver volume	Mean dose of normal liver	Dose of 700 cc (Gy)	Total dose (Gy)	Single dose (Gy)	BED_10_ (Gy)	Volume of GTV	Residual liver volume	Mean dose of normal liver	Dose of 700 cc (Gy)		
																Y/N
1	50	10	100	44.73	1471.4	8.06	11.32	55	11	115.5	13	1460	12.27	8.6	49	N
2	50	10	100	45.55	1587	8.64	5.26	49	7	83.3	83	1196	3.33	2.3	34	N
3	50	10	100	35.9	1326	13.1	7.8	54	9	102.6	187.9	964	25.89	13.1	11	N
4	50	10	100	28	1129	7.62	6.25	50	10	100	173	735	21	3	16	N
5	50	10	100	75.5	1256	5.38	2.61	48	6	76.8	14	1198	2.04	0.3	28	Y
6	49	7	83.3	39.91	1109	11.19	7	50	10	100	24.61	929	10.06	3.07	21	N
7	50	10	100	50	1589	9	6.92	55	11	115.5	13.9	1629	15.39	10.4	17	Y
8	48	6	76.8	19.1	947.2	8.12	0.4	49	7	83.3	37.47	839	15.5	1.4	33	N
9	48	6	76.8	11.2	898	9.73	3.11	54	9	102.6	16	842	5.23	2.3	7	Y
10	55	11	115.5	32.4	1384	9.64	5.3	55	11	115.5	9.64	1228	13.09	8.3	38	N
11	50	10	100	90.6	1297	11.1	7.29	49	7	83.3	81	1114	17.17	8.1	26	N
12	50	10	100	33.9	1337	8.46	4.89	54	9	102.6	22	1246	9.73	2.5	53	N
13	56	8	100.8	24.34	1984	9.07	2.12	54	9	102.6	73.8	1895	12.37	11.7	21	N
14	56	8	100.8	19.55	2184.3	9.54	0.32	54	9	102.6	15.67	1938	4.99	0.16	24	N
15	48	6	76.8	11.2	1027	7.29	1.54	50	10	100	16.13	999	8.25	1.68	11	N
16	55	11	115.5	70.36	1449	14.11	1.39	49	7	83.3	24	1166	6.25	1.21	24	N
17	50	10	100	8.9	918	7.8	2.3	48	6	76.8	37	1249	8.76	7.5	15	N
18	56	8	100.8	24.88	1306	9.83	3.67	54	9	102.6	17.6	1298	15.47	11.6	19	N
19	50	10	100	17.2	1052.7	8.02	3.16	54	9	102.6	41	1185	6.92	3.45	43	N
20	55	11	115.5	21.36	1884	8.42	1.94	54	9	102.6	53.4	1411	5.07	3.89	12	N
21	55	11	115.5	150.64	1487	12.53	11.34	50	10	100	27.8	1570	11.18	8.12	6	N
22	50	10	100	13.14	1471	7.89	1.29	49	7	83.3	9	1342	4.03	1.5	6	N
23	48	6	76.8	132.5	1636.3	13.17	9.61	50	10	100	44.64	1333.7	15.22	10.21	21	N
24	50	10	100	110	1735	17.97	14.26	50	10	100	11.2	1769	4.68	2.16	10	N

### Adverse reaction assessment

Blood test including complete blood count, biochemical parameters and coagulation function were detected every week during and after re-SBRT in all patients. Image examination were arranged every 3 months. Adverse reaction was evaluated by Toxicity criteria of the Radiation Therapy Oncology Group (RTOG) and the European Organization for Research and Treatment of Cancer (EORTC) [8]. Radiation induced liver injury (RILD) was defined as normal liver function excluding tumor progression and/or hepatitis virus replication, which had two types: classic RILD and non-classic RILD [9].

### Statistical analysis

Cumulative overall survival rates (OS), progression-free survival rates (PFS) and local control rates (LC) were calculated by Kaplan-Meier method. For comparisons between variables of two groups, the χ^2^ test and Fisher’s exact test were performed. The analysis was performed using Statistical Package for the Social Sciences software (SPSS ver. 22.0, IBM Corp., Armonk, NY) and Software for Statistics and Data Science (STATA ver.15.0, STATA Corp., College Station, TX, USA). *P*-values< 0.05 were defined as statistically significant.

## Results

By July 2019, seven patients died. Among them, four cases died of hepatic failure, and three died of upper gastrointestinal hemorrhage. All these four hepatic failure patients died beyond 10 months after radiotherapy, who didn’t meet diagnostic criteria of RILD. Two cases died of upper gastrointestinal hemorrhage patients diagnosed esophagogastric variceal bleeding (EGVB) by gastroscope. Therefore, the causes of death were related to progression or complication of liver cirrhosis.

**Fig. 1:**
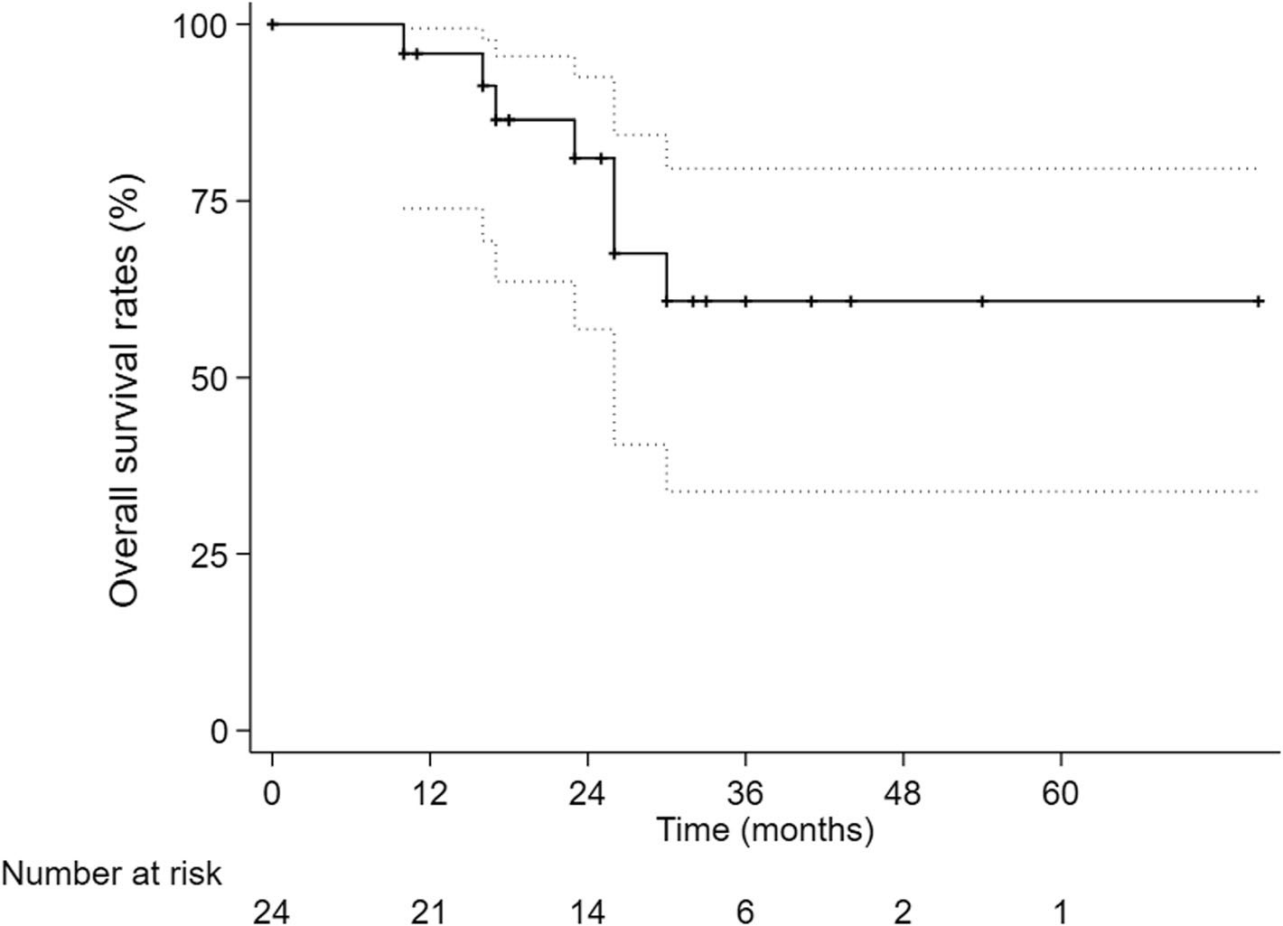
Kaplan-Meier curve of cumulative overall survival

**Fig. 2:**
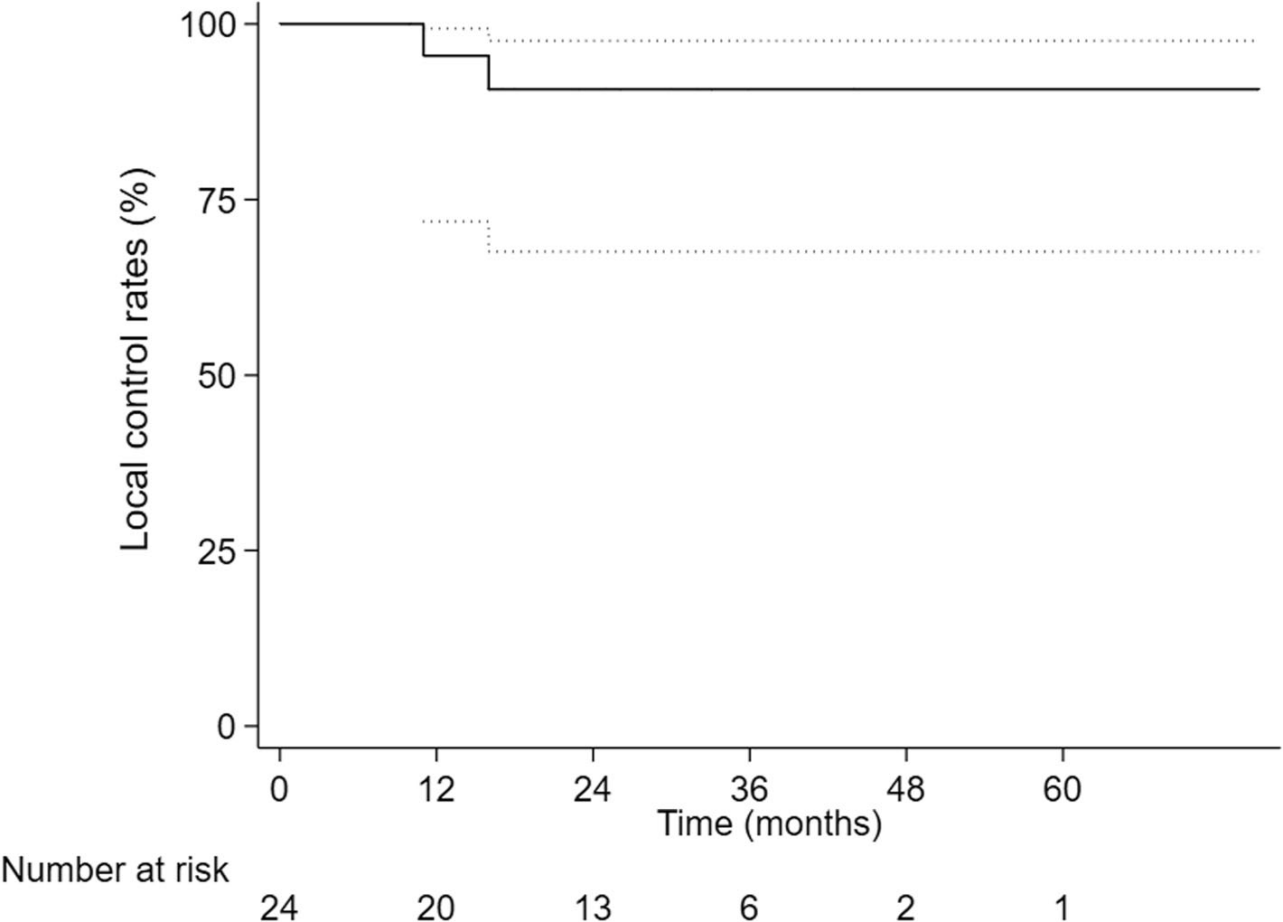
Kaplan-Meier curve of local control

**Fig. 3:**
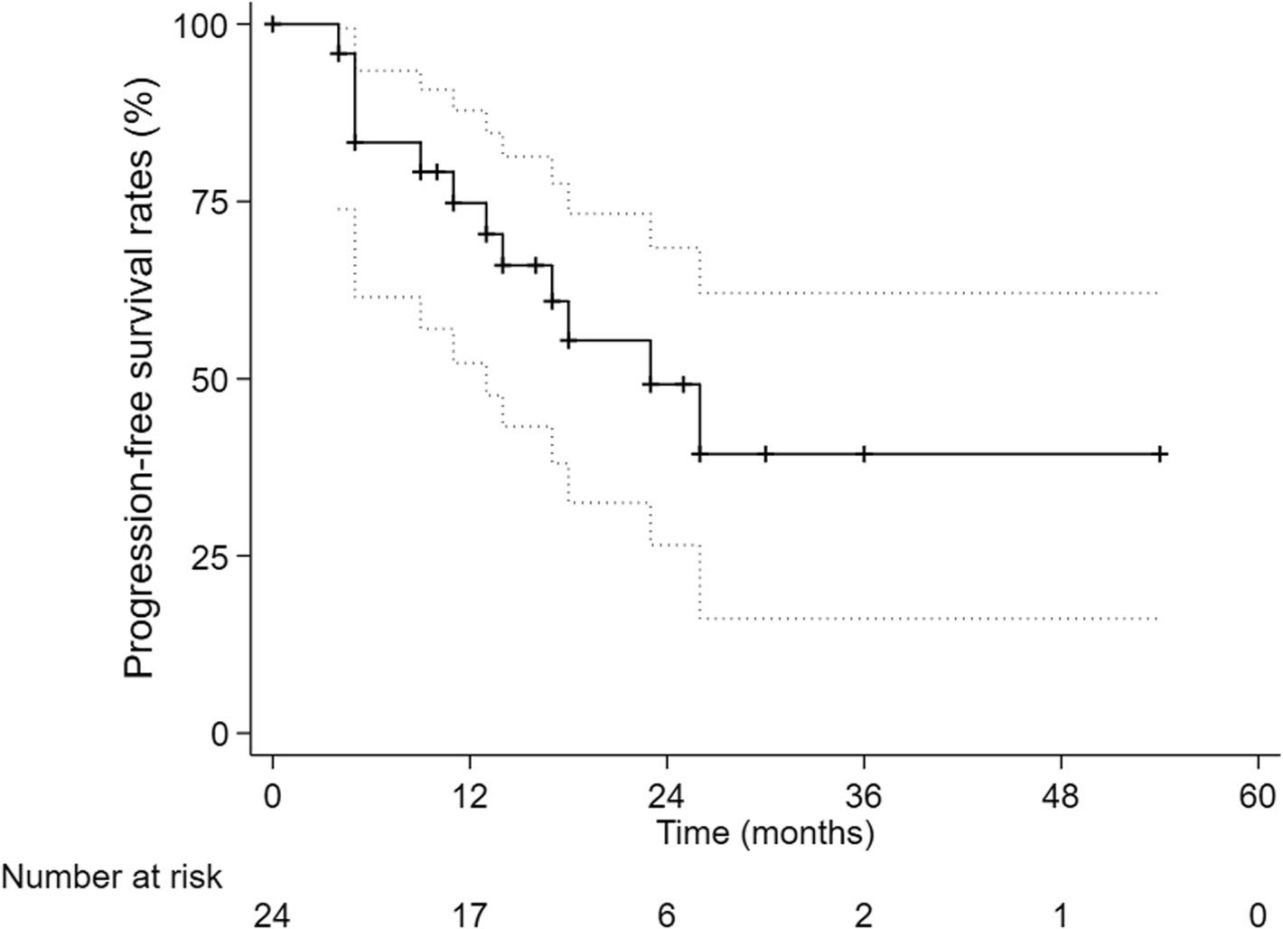
Kaplan-Meier curve of progression-free survival

**Fig. 4:**
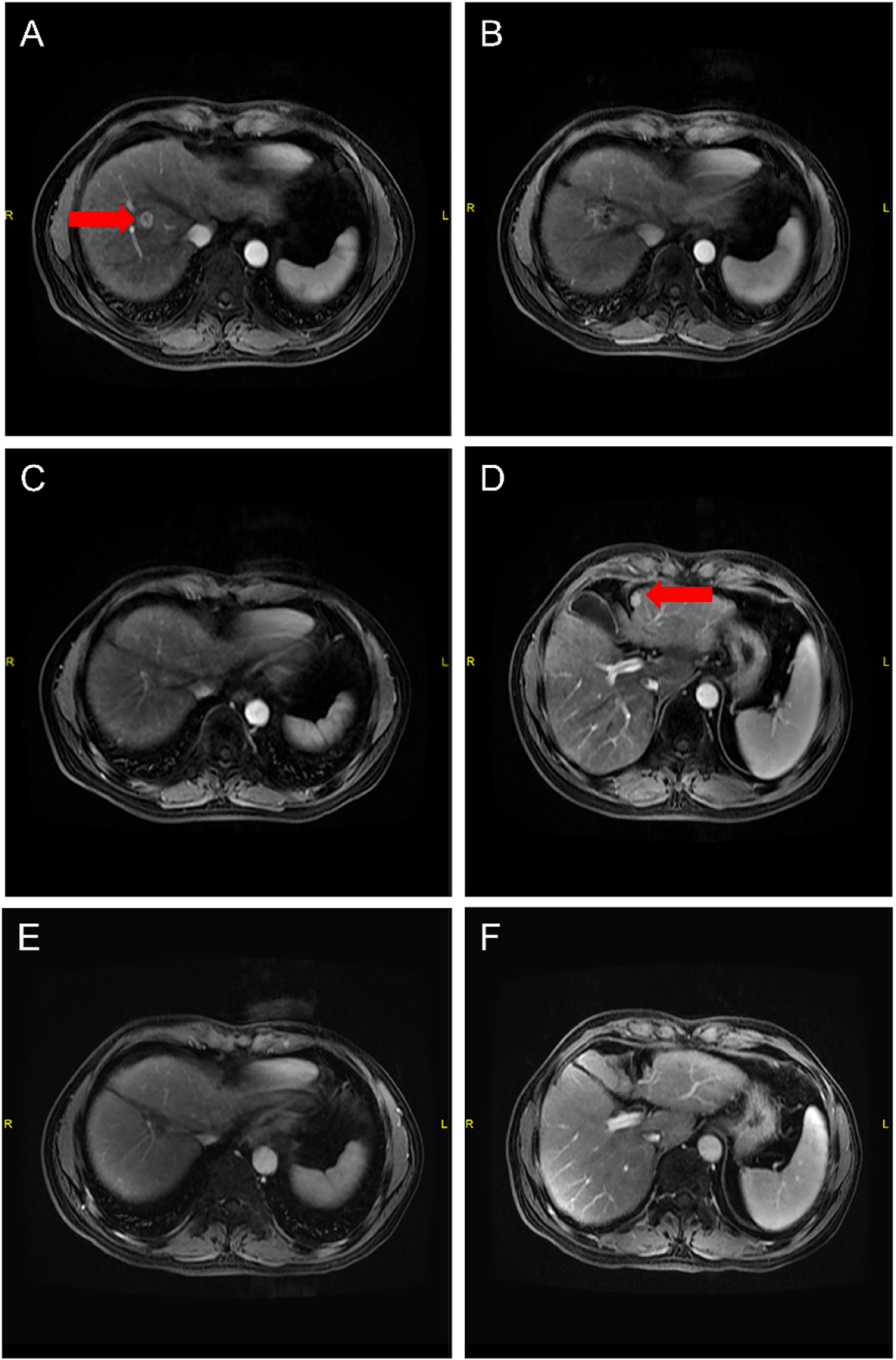
The patient who received two courses CK-SBRT, and the interval period was 26 months. October 2014: The primary abdominal MRI scan with the HCC lesion (in right liver) (**a**). Then the patient received CK-SBRT with 50Gy/5f. July 2015: MRI scan of lesion in right liver after CK-SBRT (**b**). December 2016: MRI scan of lesion in right liver showed the activity of lesion had disappeared (**c**). New lesion occurred in left liver (**d**), thereafter the patient received second-course CK-SBRT with 49Gy/7f. August 2019: MRI scan of 58 months after CK-SBRT (lesion in right liver) (**e**); MRI scan of 32 months after second-course CK-SBRT (lesion in left liver) (**f**)

**Fig. 5:**
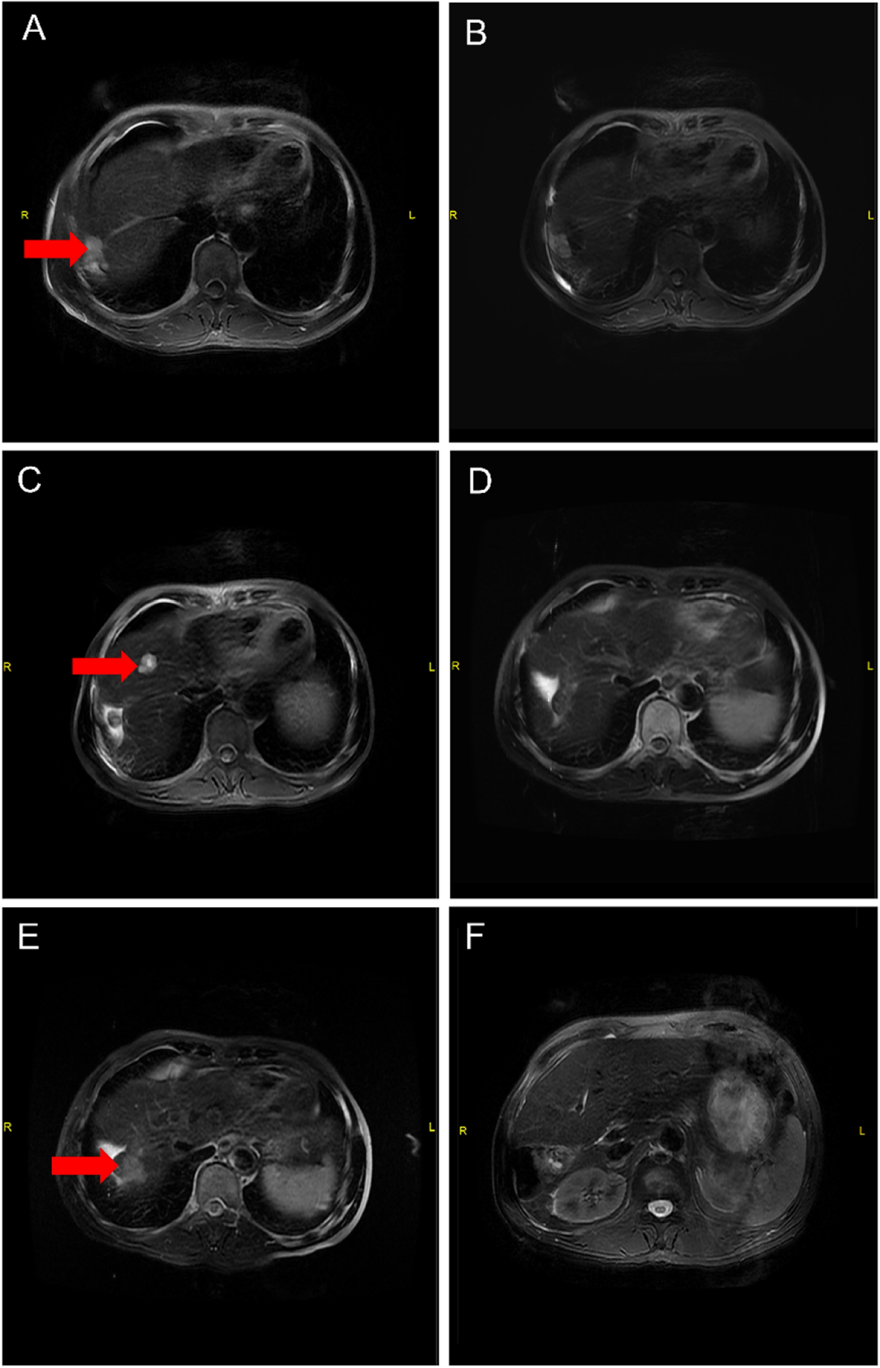
The patient who received three courses CK-SBRT. November 2014: The primary abdominal MRI scan with the HCC lesion (in right liver) (**a**). Then the patient received CK-SBRT with 56Gy/7f. July 2015: MRI scan of 8 months after CK-SBRT (lesion in right liver) (**b**). The activity of lesion had disappeared. June 2016: MRI scan of lesion right liver after CK-SBRT; New lesion occurred in liver, thereafter the patient received second-course CK-SBRT with 54Gy/6f (**c**). February 2017: MRI scan of first-treated and second-treated lesion (**d**). The activity of two lesions had disappeared. November 2017: There was an activity tumor adjacent to first-treated lesion, thereafter the patient underwent 49Gy/7f of CK-SBRT (**e**). July 2019: MRI scan of the treated area after CK-SBRT (**f**)

The 1-year, 2-year and 3-year cumulative OS were 95.8, 81.1 and 60.8% respectively (Fig. 1). The 1-year, 2-year and 3-year LC were 95.5%,90.7 and 90.7%, respectively (Fig. 2) and The 1-year, 2-year and 3-year PFS were 74.8, 49.2 and 39.4% (Fig. 3). After second-course SBRT, twelve patients experienced relapse or metastasis and thereafter three patients chose third-course SBRT. There were two cases were showed in Figures: one patient received two courses SBRT (Fig. 4); another patient received three courses SBRT (Fig. 5).

All patients finished second-course CK-SBRT. Fatigue was occurred in sixteen patients, and two patients showed Grade 2 gastrointestinal reaction in the form of anorexia with ≤15% weight loss from pretreatment baseline. Their symptoms were relieved after drug treatment. Only one patient was diagnosed RILD. The interval period between first-course SBRT and second-course SBRT in this patient was 21 months. His residual normal liver volume of second-course SBRT was 1333 cc. Before and after second-SBRT, the patient belonged Child-Pugh A5 score and Child-Pugh B7 score (total bilirubin = 35.4 μmol/L, albumin = 34 g/L), and relieved by drug therapy.

### Comparison between interval time ≥ 12 months and interval time < 12 months

**Fig. 6:**
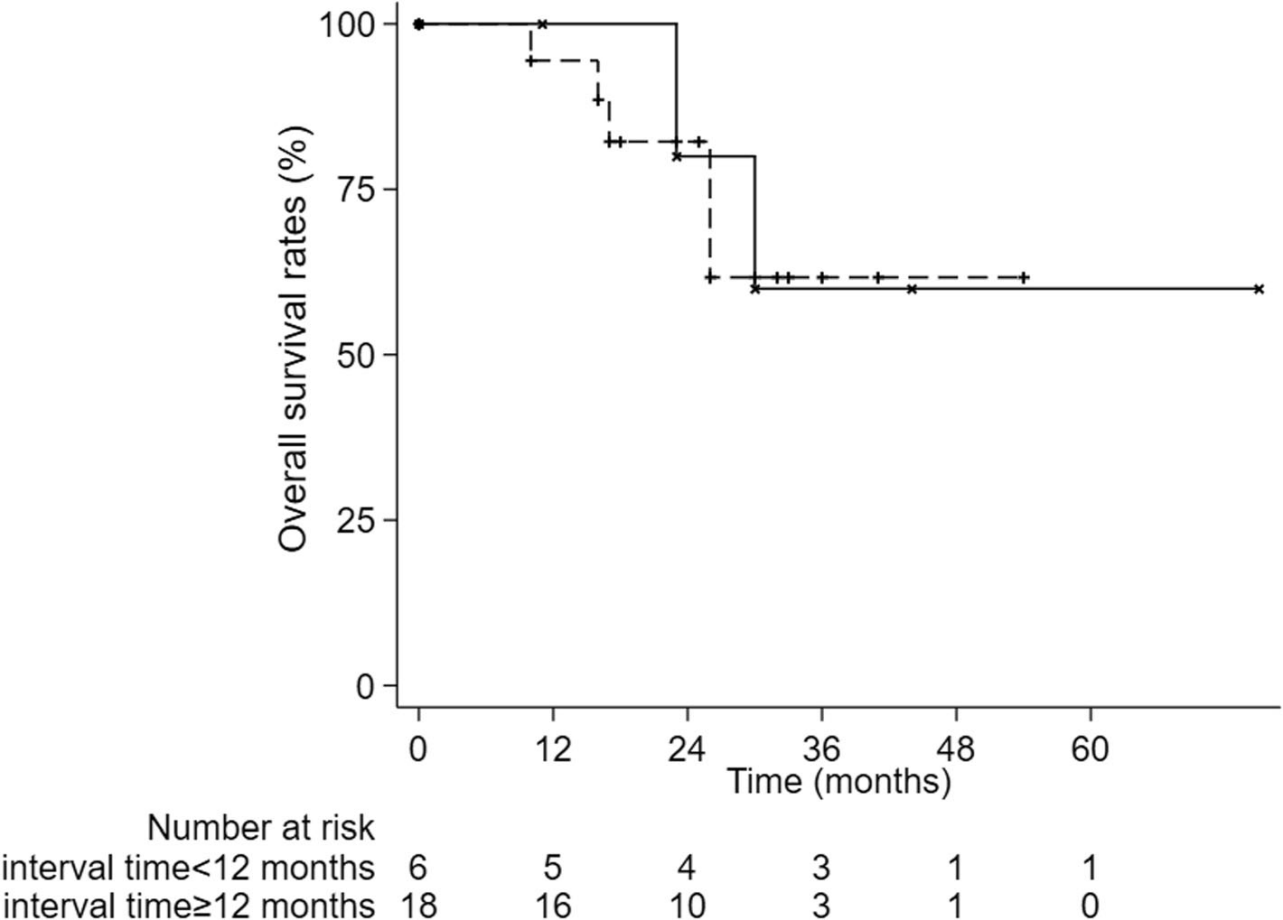
There was no significant difference in OS between the interval time < 12 months group and in the interval time ≥ 12 months (*p* = 0.913)

**Fig. 7:**
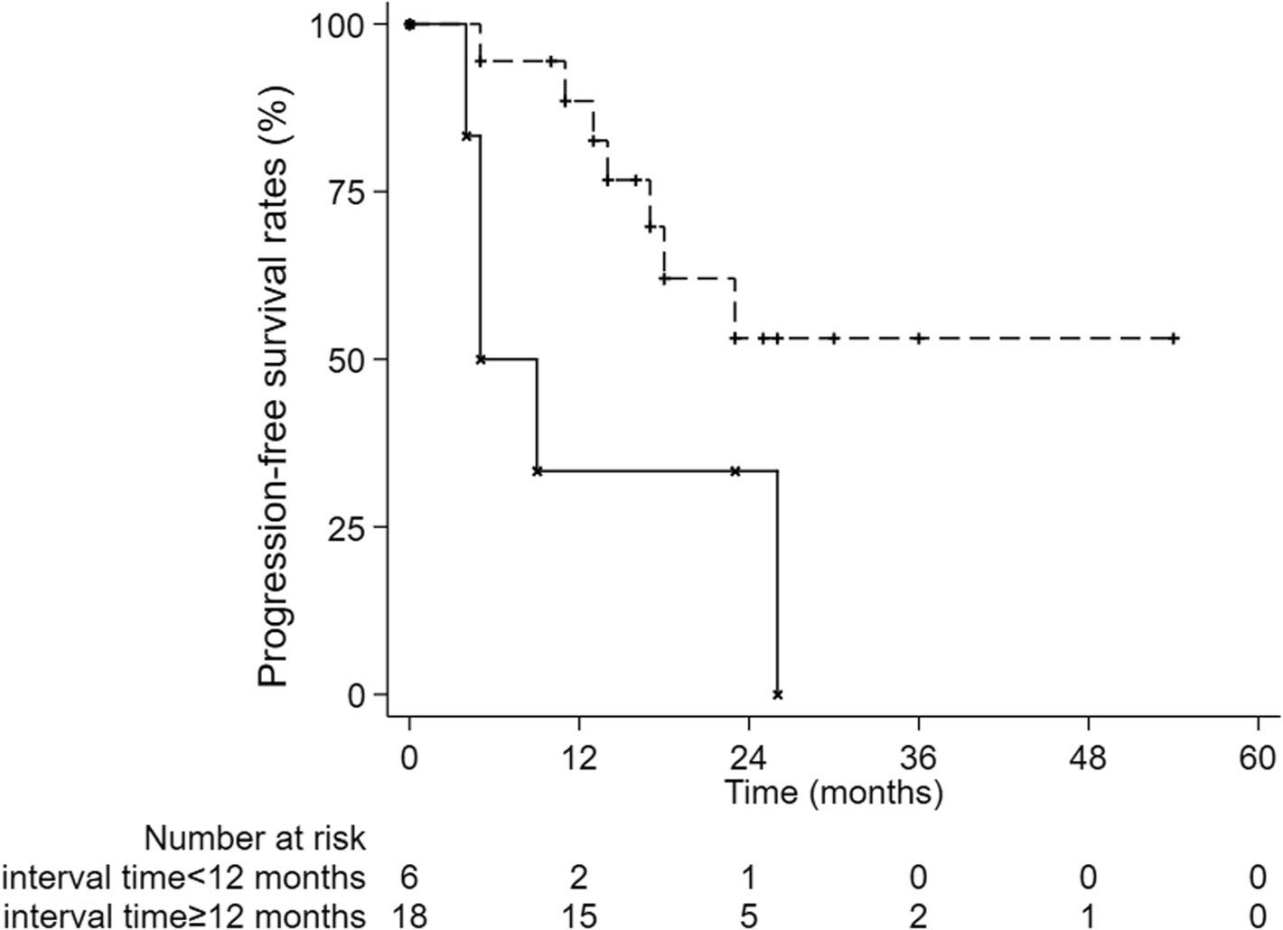
PFS was significantly higher in the interval time < 12 months group than in the interval time ≥ 12 months group (*p* = 0.030)

We divided the enrolled patients into two groups with interval time of 12 months. There was no significant difference in OS between two groups (*p* = 0.913, Fig. 6). However, the PFS was significantly higher in the interval time < 12 months group than in the interval time ≥ 12 months group (*p* = 0.030, Fig. 7).

## Discussion

SBRT has been widely applied for different stages of HCC in recent years. Previous studies showed that therapeutic effect of SBRT in treating early stage HCC was equivalent to liver resection, radiofrequency ablation (RFA), etc. Meanwhile, SBRT was also used as savage treatment after hepatic arterial chemoembolization (TACE), bridge treatment before liver transplantation and recurrent treatment after resection and RFA [10–13].

Hepatocyte has great regeneration capacity. In 1931, Higgins and Anderson built the mouse hepatocyte regeneration model, which proved residual two-third liver could restore to original size after one-third liver removing [14]. Because most of HCC patients had underlying both hepatitis virus infection and some developed to cirrhosis, their liver regeneration capacity was not likely equal to the patients without hepatitis. However, their hepatocyte could also regenerate to different degrees after treatment [15], which provided theoretical basis for treating recurrent HCC. The HCC relapse and metastasis were most important factors affecting prognosis. In addition to tumor-self, liver cirrhosis nodules were at risk of developing to new lesions. Therefore, it is important not only for primary treatment of HCC, but also subsequently treatment for recurrent HCC, which could prolong their survival.

Due to higher injury risk, especially of liver and gastrointestinal tract, repeated radiotherapy was difficult to evaluate for HCC patients. As we have known, liver is a parallel-type organ with independent functional subunits, nevertheless three-dimensional conformal radiation therapy (3D-CRT) and intensity-modulated radiation therapy (IMRT) were seldom applied for relapse HCC patients, and only a few institutions carried out repeated SBRT.

CK-SBRT has the advantage of combining respiratory synchronous tracking guides and fiducial marker tracking, which can control the precision within 1 mm, and realize precise therapy [10]. With little injury of normal tissues, occurrence rate of RILD in our study was acceptable. The OS rates in this study were lower than our previous study of naïve treatment patients [16], but the tumor size in this re-CK-SBRT study was smaller, which may be one reason for the high survival rate for re-CK-SBRT patients. Moreover, the OS in this study was higher than the result of the retrospective analysis of HCC patients who underwent secondary resection. Their result showed 1-year and 3-year OS were 88 and 67% [17]. Cheng-Hsiang Lo’s [18] reported that one- and two-year overall survival rates after CK-SBRT were 76 and 59.1%, which were lower than ours. We considered that the difference may be relate to his lower prescribed dose (median: 41Gy, range: 34-60Gy). Furthermore, in term of adverse reaction, their result showed one developed radiation-induced liver disease and three showed progression of the Child-Pugh classification after the second-course therapy. Both toxicities of our studies were generally mild and tolerable.

However, careful patient selection should not be neglected. To avoid gastrointestinal injury, we supposed the distance between lesion and gastrointestinal was more than 10 mm that was relatively safe, which based on that doses were rapid dose fall-off and very low at point 10 mm far away from PTV. Meanwhile, if the lesion was near to stomach and intestine, the smaller the lesion, the safer the treatment. The main reason was that when the tumor was small, a small collimator (5-15 mm) was selected for formulating plan who make dose decrease faster. Moreover, for decreasing the dose of previously treated and/or dose overlapping area, we usually built a shield between the PTV area and the being protect area or organ, which could significantly decrease the dose of previously treated region. In addition, the interval period between first-course SBRT and second-course SBRT was at least 6 months, which offered a certain time for liver regeneration and gastrointestinal restoration from potential damage from radiation.

It is worth mentioning that the recurrence time after first-course therapy was related to PFS after second-course therapy. Because there are only six patients whose interval time was shorter than 12 months, we need to explore large sample study to confirm this result. Moreover, we should pay more attention to these patients, shortening their review period, which may improve the diagnosis rate of recurrent HCC.

Since the sample in our study was too small, we can’t analysis the influencing factors of survival and parameters of two courses treatments. It is hard to carry out the prospective study of repeated radiation therapy, and it’s difficult to explore deeply. To summarize an objective result, we need to take into account at least the prescribed doses, interval period between two courses therapies and lesion location, but these parameters was hard to unify.

## Conclusions

It is preliminarily believed that re-CK-SBRT is an effective and safe treatment for HCC patients, but the treatment criteria should be strictly controlled. It’s worth conducting a future multi-center study with a larger number of patients to explore its feasibility and security.

## Data Availability

The datasets used and analysed during this study are available from the corresponding author on reasonable request.

## Abbreviations

AFP: Alpha-fetoprotein
BED: Biologically effective dose
CIs: Confidence intervals
CK: CyberKnife
CP: Child-Pugh
CT: computed tomography
ECOG: Eastern cooperative oncology group
GTV: Gross target volume
LC: Local control
MRI: Magnetic resonance imaging
OS: Overall survival
P: *P*-value
PTV: Planning target volume
RFA: Radiofrequency ablation
RILD: Radiation-induced liver disease
SBRT: Stereotactic body radiation therapy
